# Supplementary material for: Genome-Wide Identification, Comparison, and Expression Analysis of Transcription Factors in Ascidian Styela clava
Source: Int J Mol Sci. 2021 Apr 21;22(9):4317. doi: 10.3390/ijms22094317 (PMC8122590; doi:10.3390/ijms22094317)
Supplement: Supplementary file 1 [file ijms-22-04317-s001.zip › ijms-1094395 suppl/ijms-1094395_manuscript_supplemental_2021.pdf]

International Journal of *Molecular Sciences*

*Supplementary Materials*

# Genome-wide identification, comparison and expression analysis of transcription factors in ascidian *Styela clava*

**Jin Zhang[1,†], Jiankai Wei[1,2,3,†], Haiyan Yu[1], Bo Dong[1,2,3,*]**

[1] Sars-Fang Centre, MoE Key Laboratory of Marine Genetics and Breeding, College of Marine Life Sciences, Ocean University of China, Qingdao 266003, China; zhangjincoolcool@163.com (J.Z.); weijiankai@ouc.edu.cn (J.-K.W.); haiyanyu@ouc.edu.cn (H.-Y.Y.); bodong@ouc.edu.cn (B.D.)
[2] Laboratory for Marine Biology and Biotechnology, Qingdao National Laboratory for Marine Science and Technology, Qingdao 266237, China.
[3] Institute of Evolution and Marine Biodiversity, Ocean University of China, Qingdao 266003, China;
* Correspondence: bodong@ouc.edu.cn; Tel.: +86-0532-82032732
† These authors contributed equally to this paper.

Citation: Zhang, J.; Wei, J.; Yu, H.; Dong, B. Genome-Wide Identification, Comparison, and Expression Analysis of Transcription Factors in Ascidian *Styela clava*. *Int. J. Mol. Sci.* **2021**, *22*, 4317. https://doi.org/10.3390/ijms22094317

Academic Editors: Akira Ishihama, Igor Rogozin and Clifford Louime

Received: 15 January 2021
Accepted: 6 April 2021
Published: 21 April 2021

**Publisher's Note:** MDPI stays neutral with regard to jurisdictional claims in published maps and institutional affiliations.

**Copyright:** © 2021 by the authors. Submitted for possible open access publication under the terms and conditions of the Creative Commons Attribution (CC BY) license (http://creativecommons.org/licenses/by/4.0/).

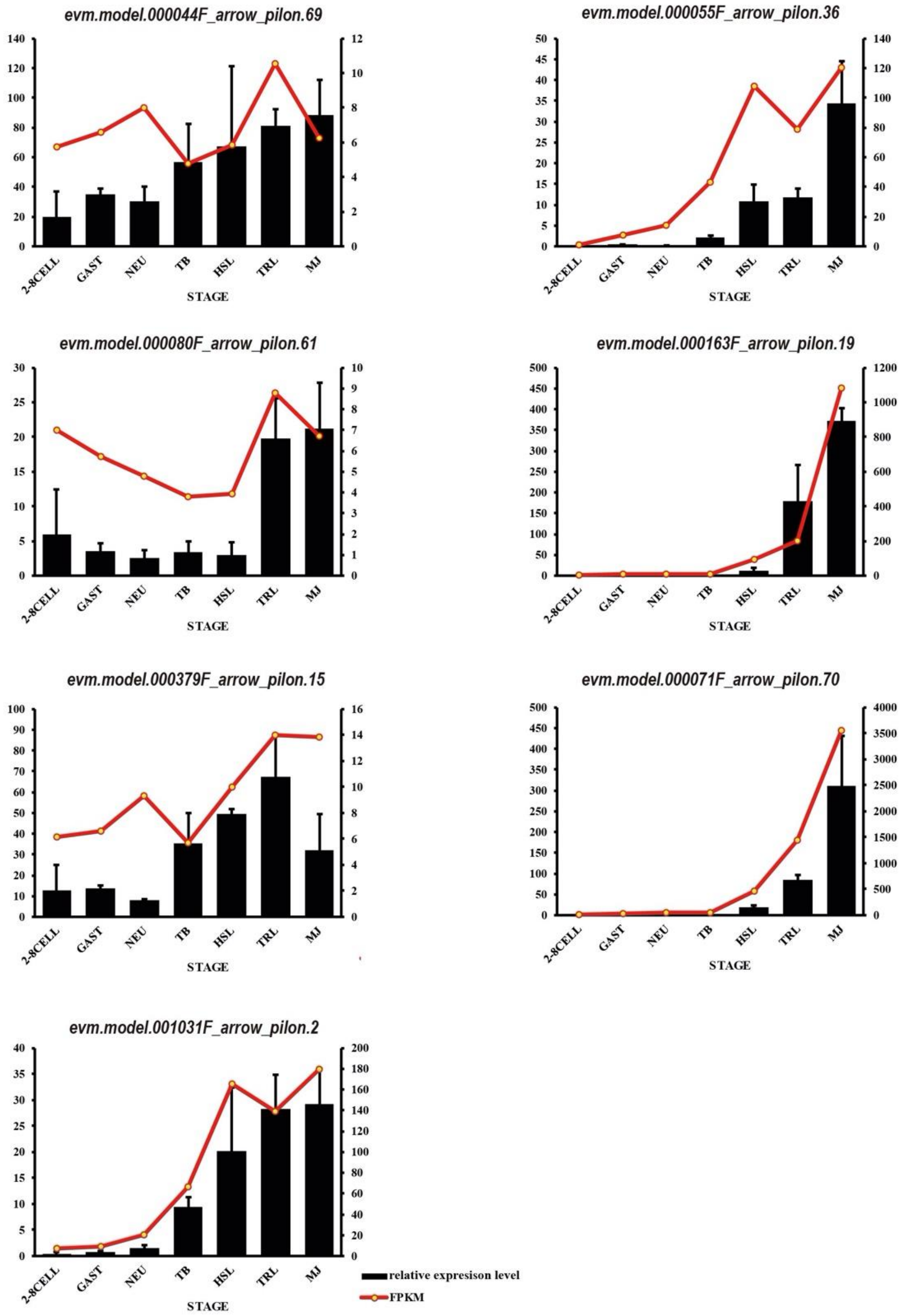


**Figure 1.** Expressions of partial TFs detected by RNA-Seq and quantitative real-time PCR (RT-qPCR). The black cylinders indicate the relative expression levels acquired though RT-qPCR. The red lines with yellow dots indicate the FPKM value from RNA-Seq. The left y-axis indicates the FPKM value and the right y-axis indicates the relative expression levels. Gene ID is shown on the top of each charts.

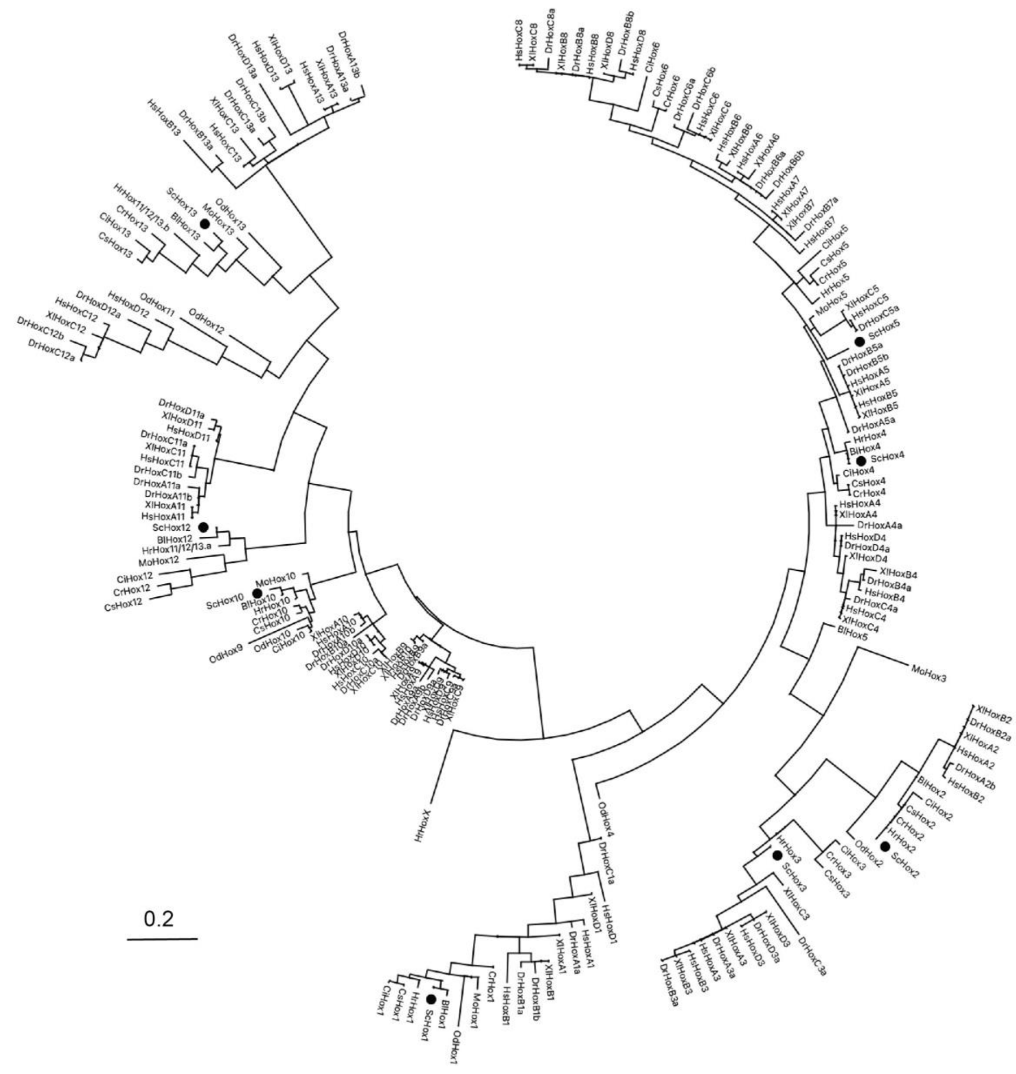


**Figure 2.** Phylogenetic tree of hox genes in *S. clava*, *C. robusta*, and *H. sapiens*. Phylogenetic tree of hox genes in *S. clava, C. robusta*, and *H. sapiens* constructed by the Maximum-Likelihood (ML) method using amino acid sequences. Black solid dots indicate *S. clava* proteins. The scale bar indicates an evolutionary distance of 0.1 amino acid substitutions per position.

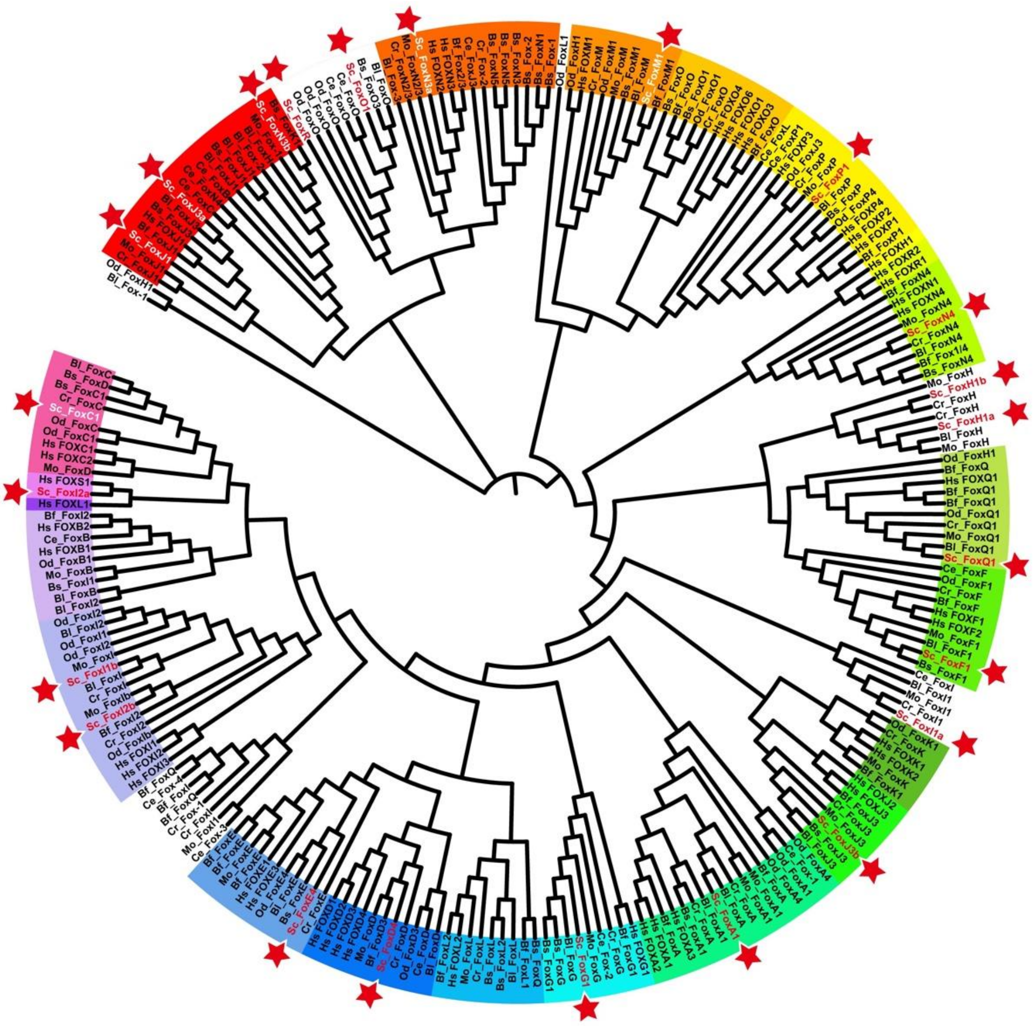

**Figure 3.** Function annotation of maternal TFs. The top 20 terms of GO enrichment analysis for maternal TFs. Y-axis indicates the GO term and x-axis indicates the Rich Factor. Size of points indicate the gene number, and the color of points indicate the q-value.

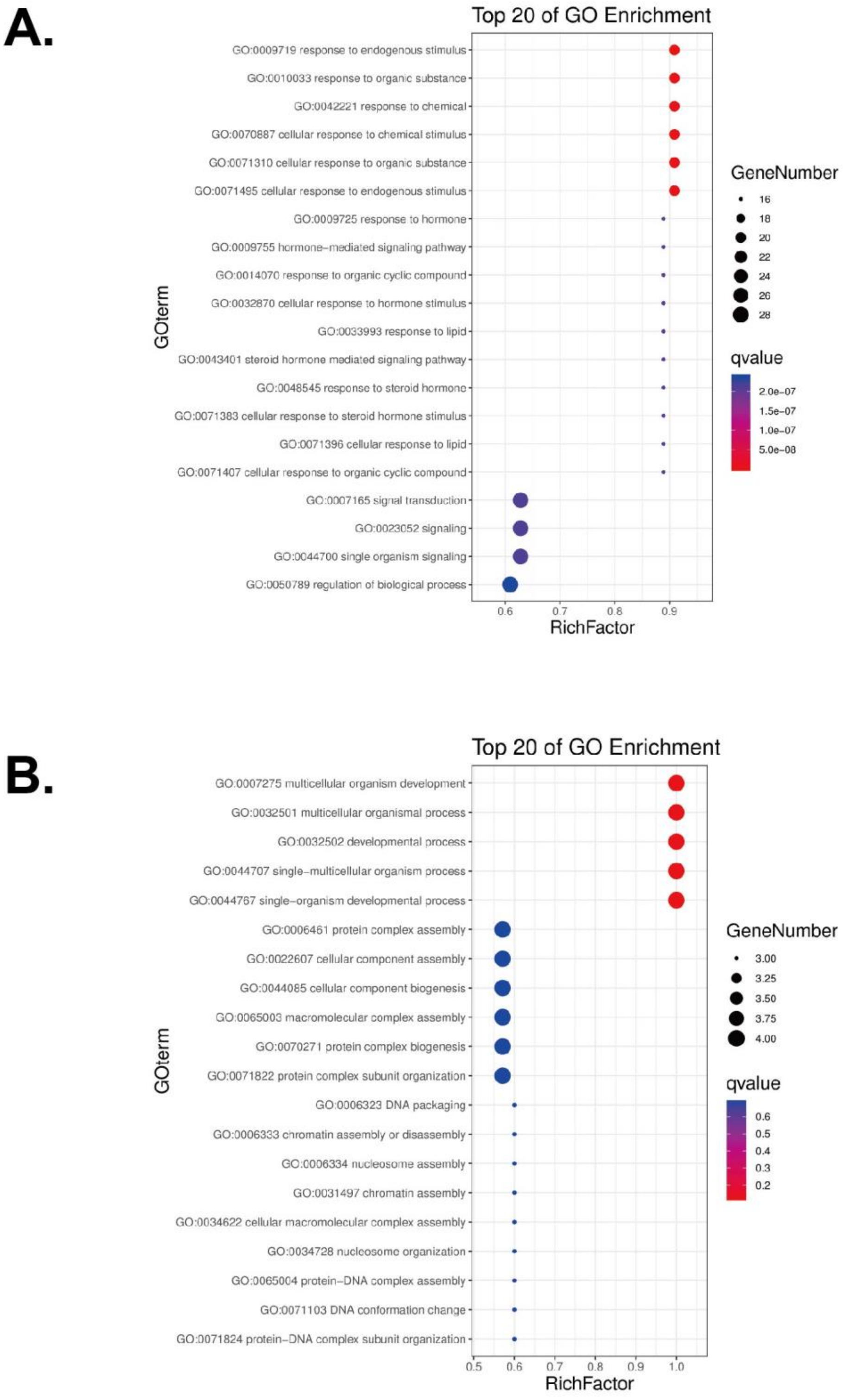


**Figure 4.** Function annotation of maternal expressed TFs and zygotically expressed (A) Function annotation of maternal expressed TFs. (B) Function annotation of zygotically expressed TFs. The top 20 terms of GO enrichment analysis for TFs. Y-axis indicates the GO term and x-axis indicates the Rich Factor. Rich Factor indicated the ratio of maternal TFs and all TFs in the GO annotation, implying the degree of enrichment. Size of points indicate the gene number, and the color of points indicate the q-value. .

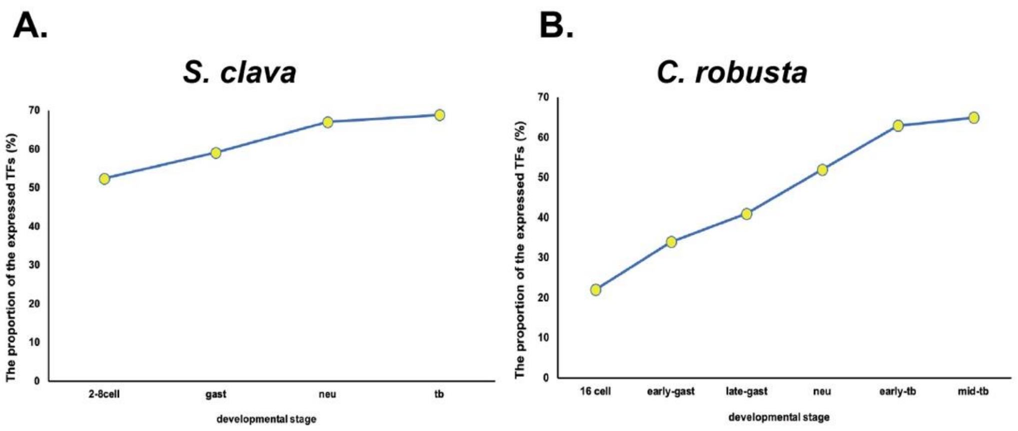


**Figure 5.** Proportion of the expressed TFs through early development of *S. clava* and *C. robusta*. (A) The proportion of the expressed TFs through early development of *S. clava*. (B) The proportion of the expressed TFs through early development of *C. robusta*. The y-axis was indicated the proportion of expressed TFs and the x-axis was indicated the developmental stage of *S. clava* and *C. robusta*.
